# A Case of Amyloid Light-Chain Amyloidosis Presenting as Colitis

**DOI:** 10.7759/cureus.103605

**Published:** 2026-02-14

**Authors:** Karpagapriya Prabakaran, Sarosh Khan, Ling Mei, Francis Edeani, Patrick Sanvanson, Priyadharshini Sivasubramaniam, Mahmoud Ali, Gokulakrishnan Balasubramanian

**Affiliations:** 1 Division of Gastroenterology and Hepatology, Department of Medicine, Medical College of Wisconsin, Milwaukee, USA; 2 Department of Pathology, Medical College of Wisconsin, Milwaukee, USA

**Keywords:** amyloidosis, colitis, colonic amyloidosis, gastrointestinal amyloidosis, lower gastrointestinal bleeding

## Abstract

Gastrointestinal amyloidosis is a rare but often a manifestation of an advanced form of systemic amyloidosis that carries a poor prognosis. Its presentation may mimic ischemic bowel disease, inflammatory bowel disease, celiac disease, or protein-losing enteropathy, among others. Often, varied presentations make it difficult to diagnose. Hence, a careful and systematic approach with an aggressive treatment strategy is needed for prompt diagnosis and treatment of the fatal condition. Here, we report a fatal case of systemic amyloidosis (amyloid light-chain (AL) amyloidosis) with its initial presentation including gastrointestinal symptoms and subsequent rapid clinical deterioration due to worsening renal function and evidence of pericardial effusion.

## Introduction

Amyloidosis is a rare disorder characterized by the deposition of misfolded amyloid proteins, which can occur in systemic or localized forms. It can be classified into major systemic types, including amyloid light-chain (AL) amyloidosis (associated with plasma cell dyscrasia), amyloid A amyloidosis (AA) (associated with chronic inflammatory disorders), and transthyretin amyloidosis (ATTR) (associated with cardiac and neurologic involvement), as well as localized or organ-specific types such as atrial natriuretic factor amyloidosis (isolated atrial) and Acal amyloidosis (calcitonin‑related). Approximately 3% of patients with amyloidosis have gastrointestinal (GI) involvement. Depending on the site of production and deposition of the amyloidogenic precursor proteins, the disease may be limited to the GI tract as a localized process or represent part of systemic involvement [1]. Because of its variable and nonspecific manifestations, including acid reflux, abdominal pain, bloating, GI bleeding, diarrhea, nausea, vomiting, dysphagia, and pseudo-obstruction, diagnosis can be challenging [1-3]. More than half of patients may present with GI bleeding due to ischemia, ulcers, erosions, or submucosal hematomas [4,5]. As a result, these patients often undergo extensive diagnostic evaluation, which can delay recognition of the underlying condition. The clinical presentation and prognosis depend on the location, quantity, and type of amyloid protein deposition. Here, we describe a rare case of biopsy‑proven AL amyloidosis that initially presented with lower GI bleeding, highlighting the diagnostic challenges and the importance of tissue-based confirmation.

## Case presentation

**Figure 1:**
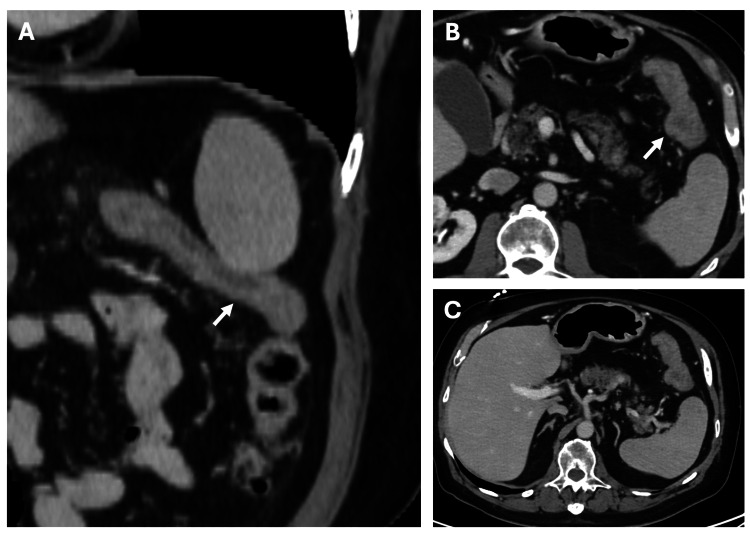
CT angiography of the abdomen and pelvis with (A) coronal and (B and C) axial views revealing diffuse wall thickening with mild pericolonic fat stranding involving the splenic colonic flexure (indicated by arrows), concerning for acute ischemic colitis.

**Figure 2:**
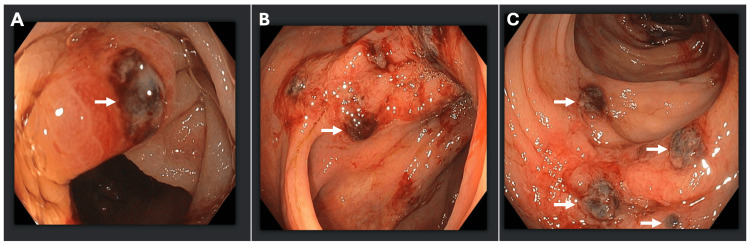
(A-C) Colonoscopy images illustrating segmental ulcerations with surrounding mucosal edema and clot (white arrows).

**Figure 3:**
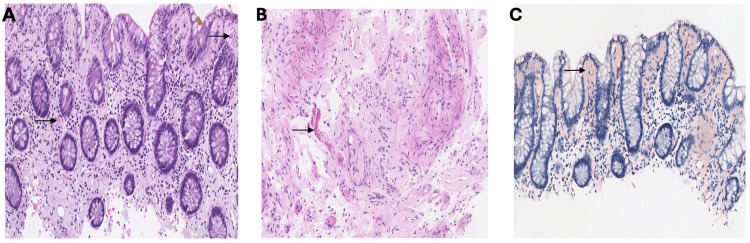
(A) Colonic biopsy showing accumulation of amorphous eosinophilic material in the subepithelial lamina propria and around blood vessels (black arrows). (B) Submucosal vessels with accumulation of amorphous eosinophilic material in its walls (black arrow). (C) Amyloid deposits are highlighted by Congo red stain seen as orange material (black arrows).

A 75-year-old male presented to the emergency room with painful rectal bleeding. His past medical history was significant for diverticulosis, thoracic myelopathy due to a T12-L1 lesion, hypertension, type II diabetes mellitus, and hypothyroidism. He denied any prior history of intra-abdominal surgeries. The patient had developed diarrhea and abdominal cramping three days before presentation. He had also been experiencing progressive fatigue and shortness of breath with exertion for the past couple of months before presentation. His physical examination was unremarkable except for positive stool guaiac testing performed at the bedside. Laboratory work was significant for elevated creatinine with nephrotic-range proteinuria. CT of the abdomen and pelvis revealed diffuse wall thickening with mild pericolonic fat stranding involving the splenic colonic flexure, concerning for acute ischemic colitis (Figure 1). During hospitalization, the patient received supportive care for the management of colitis and acute kidney injury. The patient underwent a colonoscopy, which revealed segmental ulceration with surrounding mucosal edema and blood clots, particularly in the hepatic and splenic flexures, concerning for ischemic colitis. Additionally, incidental diverticulosis of the sigmoid colon was noted (Figure 2). Biopsy results revealed findings consistent with the diagnosis of amyloidosis, indicating AL (kappa-type) amyloid deposition as confirmed by the Congo red stain (Figure 3). Detailed biopsy results are presented in Table 1. The patient was subsequently referred to the hematology/oncology service. Further workup, including serum and urine protein electrophoresis, echocardiogram, bone marrow and fat pad biopsy (Table 1) was performed, and he was diagnosed with AL-type amyloidosis with systemic involvement characterized by pericardial effusion causing cardiac and renal failure (Mayo Stage Stage IV, Cardiac Stage 3b).

**Table 1:** Results from studies conducted during the patient’s clinical course.

Test	Result
Colon biopsy	Accumulation of amorphous eosinophilic material in the subepithelial lamina propria and around blood vessels. Congo red stain highlighted the amyloid deposits and exhibited apple-green birefringence under polarized light. These amyloid deposits stained gray with trichrome stain. Liquid chromatography tandem mass spectrometry detected a peptide profile consistent with AL (kappa)-type amyloid deposition
Serum protein electrophoresis	Monoclonal bands present; IgA kappa (in the beta region) and free kappa
Urine protein electrophoresis	Monoclonal bands present; IgA kappa monoclonal protein in urine
Echocardiogram	Left ventricle ejection fraction 73%; moderate pericardial effusion with no evidence of tamponade
Bone marrow biopsy	Plasma cell neoplasm involving a normocellular bone marrow with trilineage hematopoiesis and a minute focus of amyloid deposition; minute CD5(+), CD10(-) monoclonal B-cell population identified by flow cytometry
Fat pad biopsy	Fibroadipose tissue with focal, minimal amorphous eosinophilic depositions, consistent with amyloid deposition (confirmed by Congo red stain); amyloid depositions showed apple green birefringence under polarized light

The patient was started on systemic chemotherapy with daratumumab, cyclophosphamide, bortezomib, and dexamethasone. Subsequently, he was readmitted with abdominal pain and elevated lactic acid, concerning for ischemic bowel injury (lactic acid, 4.6 mmol/L; normal, 0.5-2.0 mmol/L). Because of worsening clinical status, he underwent urgent laparoscopy that showed hyperemic small bowel with no evidence of bowel necrosis. His postoperative course was complicated by ileus, deteriorating renal function, coagulopathy, and hepatic dysfunction. Due to multi-organ involvement, goals of care were discussed with the patient and his family, and it was decided to pursue comfort care measures with hospice.

## Discussion

We presented a case of systemic amyloidosis initially presenting with diarrhea and lower GI bleeding. Systemic amyloidosis is more prevalent than localized amyloidosis [2]. In a 13-year retrospective review by Cowan et al., 76 patients with GI amyloidosis were identified; of these, 79% had systemic involvement, while only 21% had localized disease [1]. In our case, further workup revealed amyloid involvement of both the kidney and heart, confirming systemic involvement.

GI bleeding can develop in 57% of cases with systemic amyloidosis and is attributed to mucosal erosions and ulcerations, as noticed in our case [5]. Diarrhea is also a frequent manifestation in patients with GI amyloidosis, driven by mechanisms such as malabsorption, dysmotility, intestinal inflammation, and bacterial overgrowth [1,2].

Histopathological examination of the colon biopsy of our patient revealed AL (kappa) amyloid deposition. While various amyloid proteins can be identified in GI biopsies, AL amyloidosis remains the most commonly reported in systemic cases [1,6,7]. In the observational study of 542 patients with GI amyloidosis, the highest yield for AL(κ type) and AA amyloidosis was noted with the biopsies from the upper gastrointestinal tract (stomach and duodenum), while AL (λ type) and ATTR amyloidosis were noticed to have preferential involvement in the rectum and colon [6]. Additionally, AA amyloid commonly deposits in the lamina propria, whereas AL amyloid is more frequently identified in the submucosa and muscularis mucosa [3,6,8]. Conversely, our patient with AL (κ type) amyloidosis had lamina propria deposits with colon involvement.

Although GI involvement in amyloidosis may contribute to the morbidity of the disease, it does not contribute to its mortality. Mortality is typically related to cardiac or renal complications. One study suggested that patients with AL amyloidosis having GI involvement may be associated with decreased survival, though the findings were not statistically significant [9]. However, another study reported that the median overall survival in this group was not statistically significant during follow-up, reflecting a variable prognosis [1]. Often, these are retrospective with a limited sample size, which makes it challenging to know the proper prognosis.

## Conclusions

This case highlights the diagnostic challenge posed by colonic amyloidosis. It reinforces the need to consider amyloidosis in elderly patients who present with unexplained GI bleeding or chronic diarrhea, often associated with co-existent systemic conditions such as cardiac failure, hypercalcemia, or renal failure. Early confirmation of the condition through biopsy with Congo red stain, along with an appropriate assessment for subtype and systemic involvement, is crucial for determining the long-term prognosis.
